# Multi-professional clinical medication reviews in care homes for the elderly: study protocol for a randomised controlled trial with cost effectiveness analysis

**DOI:** 10.1186/1745-6215-12-218

**Published:** 2011-10-05

**Authors:** James Desborough, Julie Houghton, John Wood, David Wright, Richard Holland, Tracey Sach, Sue Ashwell, Val Shaw

**Affiliations:** 1 School of Pharmacy, University of East Anglia, Norwich Research Park, Norwich, Norfolk, NR4 7TJ, UK; 2 Norwich Medical School, University of East Anglia, Norwich Research Park, Norwich, Norfolk, NR4 7TJ, UK; 3 NHS Cambridgeshire, Hunts Area Offices, California Road, Huntington, Cambridgeshire, PE29 1BN, UK; 4 Cambridge University Hospitals NHS Foundation Trust, Hills Road, Cambridge, CB2 0QQ, UK

## Abstract

**Background:**

Evidence demonstrates that measures are needed to optimise therapy and improve administration of medicines in care homes for older people. The aim of this study is to determine the clinical and cost effectiveness of a novel model of multi-professional medication review.

**Methods:**

A cluster randomised controlled trial design, involving thirty care homes. In line with current practice in medication reviews, recruitment and consent will be sought from general practitioners and care homes, rather than individual residents. Care homes will be segmented according to size and resident mix and allocated to the intervention arm (15 homes) or control arm (15 homes) sequentially using minimisation. Intervention homes will receive a multi-professional medication review at baseline and at 6 months, with follow-up at 12 months. Control homes will receive usual care (support they currently receive from the National Health Service), with data collection at baseline and 12 months. The novelty of the intervention is a review of medications by a multi-disciplinary team. Primary outcome measures are number of falls and potentially inappropriate prescribing. Secondary outcome measures include medication costs, health care resource use, hospitalisations and mortality.

The null hypothesis proposes no difference in primary outcomes between intervention and control patients. The primary outcome variable (number of falls) will be analysed using a linear mixed model, with the intervention specified as a fixed effect and care homes included as a random effect. Analyses will be at the level of the care home. The economic evaluation will estimate the cost-effectiveness of the intervention compared to usual care from a National Health Service and personal social services perspective.

The study is not measuring the impact of the intervention on professional working relationships, the medicines culture in care homes or the generic health-related quality of life of residents.

**Discussion:**

This study will establish the effectiveness of a new model of multi-professional clinical medication reviews in care homes, using novel approaches to recruitment and consent. It is the first study to undertake an examination of direct patient outcomes, together with an economic analysis.

**Trial Registration:**

ISRCTN: ISRCTN90761620

## Background

This trial is concerned with the management of medicines in care homes. In 2001 there were 528,000 registered beds in 27,480 general residential and nursing homes in the UK and by 2020 this is predicted to increase by 23% [1]. General Practitioners (GPs) have principal responsibility for the medical care of care home residents and, owing to the relatively high morbidity in this population, the size of the care home population has a major impact on their overall workload [2]. Care home residents are commonly the most infirm members of the primary care community with 82% of older people in care homes having long-standing illness and 48% having two or more chronic conditions [3]. The high level of morbidity in this population is associated with a high level of prescription medicines, with care home (residential and nursing) residents receiving an average of seven medicines [4,5]. A report by the National Care Standards Commission (NCSC) in 2004 suggested that care providers seek greater involvement of pharmacists in medicines management issues and that primary care should consider commissioning reviews of prescribing practice in care homes after they found that only 44% of care homes for older people met the national minimum standards on medication [3].

Evidence demonstrates that medicines management of residents in care homes for older people could be significantly improved [6]. Medication reviews for such patients have identified high proportions of patients receiving sub-optimal therapy, with the main medication error being the continuation of medication that is no longer required [7,8]. Other problems have been identified, such as prescribing medication to counteract side effects of other medication [9], or prescribing without monitoring [10] - the exact extent of these problems is unknown. Poor medicines management leads to therapeutic failure and influences the likelihood of adverse events, for example, increasing number of falls [11] or, in the case of certain drugs used in patients with dementia, reducing survival [12]. There have been a number of calls for measures to optimise therapy and to improve the administration of medicines in this context [13], with a multi-disciplinary approach to care recommended [14].

Although studies of pharmacist medication reviews in primary care have demonstrated pharmacists' ability to identify and resolve drug-related problems and reduce prescribing [15-17], these results have not been generalisable as they tended to use a small number of pharmacists. Studies that have used a larger number of pharmacists are usually confounded by lower quality relationships with prescribers and have failed to identify significant improvements in clinical outcomes [18-22], with one study identifying an increase in hospital admissions [23].

Pharmacist-led medication reviews in care homes have also demonstrated the ability to reduce the number of prescribed medicines, but failed to detect significant change in morbidity or mortality [7,24]. Zermansky *et al. *performed a Randomised Controlled Trial (RCT) on 661 care home residents [5]. One clinical pharmacist performed the medication reviews and made recommendations to the GP. After six months follow-up the number of medication changes in the intervention group had significantly increased and the number of falls was reduced, but no other patient benefits were detected.

Very few RCTs of pharmacist-led medication reviews in the UK have included an economic evaluation. The aim of most pharmacist medication review studies has been to improve patient treatment by altering medication, thus medication costs have been included in three RCTs which demonstrated favourable prescribing outcomes [5,15,17]. Furniss *et al's. *RCT in nursing homes considered the costs (using local figures) of all primary and secondary care contact for each resident. However, the authors reported that formal economic evaluation was not possible as healthcare professionals often visited patients not included in the study during their visits [7].

It is evident from the research available that pharmacists working in isolation have limited impact on patient orientated outcomes in care homes and have failed to demonstrate cost effectiveness. The aim of this RCT is to determine the clinical and cost effectiveness of a multi-professional team approach to medication reviews in care homes.

This novel model of medication review was developed by a Primary Care Trust (PCT) Medicines Management Team (MMT) in collaboration with GPs and after consultation with geriatricians. Within the NHS, PCTs are responsible for the care of approximately 500,000 patients within a local area, assessing need and commissioning care. The vast majority of care is commissioned from independent providers such as GP practices, Community Pharmacists, Dentists and other healthcare providers. All PCTs include a MMT who are responsible for managing prescribing budgets and prescribing systems, together with supporting primary care providers. Therefore, many PCTs employ pharmacists and technicians to work with GP practices to support prescribing and this often involves reviewing patients' medication. This activity is usually completed independently by the MMT and then interventions are agreed with the prescriber at a later date. By improving the management of medicines, the new model under evaluation is designed to deliver better health outcomes for care home residents and enhance the use of healthcare resources. The uniqueness of the model is the multi-professional team approach, involving pharmacists, pharmacy technicians and care home staff, as well as the GP(s) responsible for the medical care of residents. The multi-disciplinary team meet together to review and discuss the medications of care home residents. This model has the potential for wider implementation, but the effectiveness of this approach requires evaluation.

The clinical medication review involves consideration of patients' medical history, recent blood test results and current medications. Prior to the meeting the pharmacist will review patient data and prepare a list of recommended actions. The latter will be considered at the review meeting, with the pharmacist drawing on the expertise of the GP and care home staff. The medication review is designed to identify the high instances of inappropriate prescribing in care home residents[25], reduce the number of medication errors [4] and increase the frequency of monitoring. The majority of drug-related problems in care home residents relate to psychoactive medication [26] and therefore stopping these medications, combined with the other benefits of a clinical medication review in this population, is predicted to reduce falls [27]. The review meeting also offers the opportunity for generic medicines management issues at the home to be raised.

The objectives of the RCT are to determine the impact of the multi-professional medication review service (MMRS) on the number of falls and potentially inappropriate prescribing (number of drugs which match the Screening Tool of Older Persons Prescriptions (STOPP) criteria [28] at each data collection point). The trial will also examine the impact on medication costs, utilisation of health and social care resources, and mortality. The primary analysis is a direct comparison of the two groups: intervention and control. Analyses will essentially be at the level of the care home rather than the patient (see section entitled Clinical analysis).

## Methods

### Trial design

**Figure 1:**
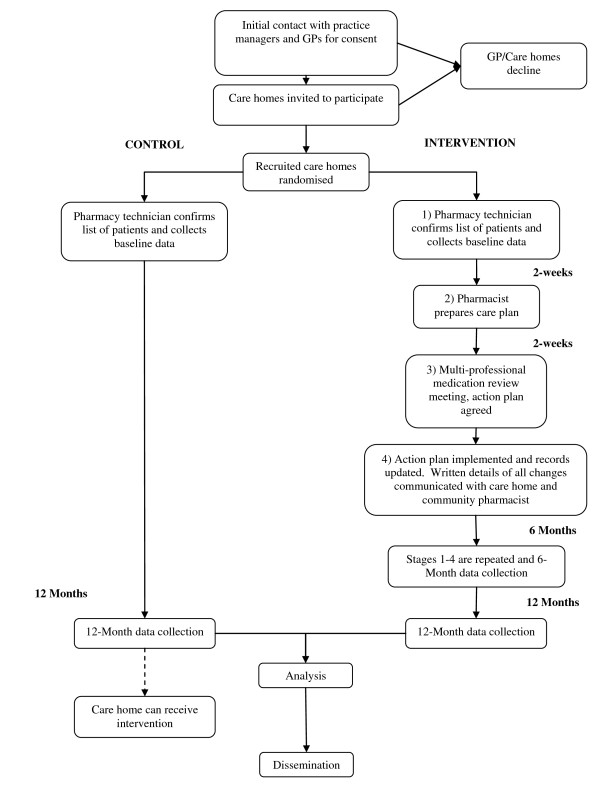
Flow chart of trial design.

A cluster randomised controlled trial will be used and a flow chart outlining the study design appears in Figure 1. The project will involve care homes for older people (average age > 65 years) registered with a GP in the local area. Thirty care homes will be recruited to the trial: 15 in the intervention arm and 15 in the control arm. Intervention homes will receive a MMRS at baseline and six months, with final data collection at 12 months. Control homes will receive usual care (support they currently receive from the National Health Service (NHS)) with data collection at baseline and at 12 months. Following final data collection at the control homes, a MMRS can be implemented.

The study has ethical approval from the NHS Norfolk Research Ethics Committee (REC reference 09/H0310/96).

### Recruitment

In the first instance, the GP practices responsible for the medical care of residents will be contacted by letter and accompanying information sheet to advise them of the study. It is expected that support and endorsement of the study by the Chairs of the Locality Prescribing Groups and the Primary Care Research Network will assist in promoting the study. A follow-up phone call from a researcher will establish expressions of interest and arrange a face-to-face meeting to explain the study in more detail.

Following agreement from GPs to include the MMT in the service responsible for the clinical care of care home residents, care homes will be approached for their consent. It is expected that care homes will be more willing to consider participation if they know that the GP practices have already given their consent to taking part in the study. A similar process of contact will be used, i.e. initial contact by letter and information sheet, with follow-up phone call and face-to-face meeting. Consent will be sought at the care home management level, rather than individual resident level. The process of obtaining consent from GPs and care home managers is in line with current practice for medication reviews in care homes.

The intervention is at the level of the home and, following standard practice, all residents in the home will have their medication reviewed. A leaflet will be given to residents (or their relatives) informing them about the study.

### Eligibility of care homes

There are more than 130 care homes in the PCT area, but after all exclusions it is predicted there will be approximately 65 care homes eligible for participation.

In order to take part the care homes must be for older people (average age >65 years) and have been registered with the Care Quality Commission (CQC) for at least six months. Care homes specifically for people (of all ages) with learning disability, sensory impairment, mental health problems, physical disabilities and alcohol dependence will be excluded. Care homes will also be excluded if they have received a medication review service from the PCT in the last 6 months, if they receive the services of a community geriatrician or if they are subject to investigation of the safeguarding of vulnerable adults.

As consent is being sought at the care home level, residents who self-medicate will be excluded. Those residents who are in the home for respite care will also be excluded.

### Allocation to treatment

Care homes will be segmented according to their resident mix (three categories: care home, care home with nursing, care home with residential and nursing beds) and size (i.e. number of beds - again three categories: small, medium and large). Recruitment will then proceed in 'waves'.

In the first wave a relatively homogeneous block of homes with approximately 30 residents will be approached as this represents the optimum number of residents for delivery of the intervention. For the second and subsequent waves, the homes to be approached will be decided purely on the basis of facilitating recruitment. For practical (i.e. workload) reasons, consenting homes will be allocated to intervention or control sequentially after consent is obtained using minimisation. This strategy has the advantage of achieving approximate balance between treatment groups with respect to stratifying factors (here resident mix and size) whilst allowing the practical advantage of being able to make allocations sequentially as homes are recruited, rather than having to wait for blocks to accumulate. Recruitment will cease once approximately 900 residents have been recruited.

### Sample size estimation

Previous research suggests that medication review services can reduce the average number of falls per patient over a 6 month period by 0.59 (confidence interval: 0.49 to 0.70), with a variance per patient of four [5]. This study was conducted in a similar setting and therefore we estimate the baseline frequency of falls to be similar (approximately 1 per resident per six months). Using the lower end of this confidence interval, 261 patients in each group would be required to detect this difference with 80% power and at the 5% level of significance. Assuming the intra-cluster correlation coefficient is 0.02 then in an average home (30 residents) this sample size needs to be multiplied by 1.58 [29] (the design effect allowing for clustering). Thus a total of 824 patients (412 in each group) will need to be recruited from approximately thirty homes. In recruiting 30 homes with an average of 30 residents this will allow for 10% losses to follow-up.

### Intervention

This is the first study that brings together a multi-disciplinary team to review the medications of care home residents in a face-to-face meeting where they are able to draw on, and benefit from, one another's expertise. The intervention is a multi-professional medication review meeting involving a clinical pharmacist and pharmacy technician from the PCT MMT, care home staff and GP(s) responsible for the medical care of residents. All pharmacists (n = 4 to 8) and technicians (n = 4 to 8) participating in this study have previous experience of reviewing the medication of care home residents. It is hypothesised that this model of medication review service has greater potential to deliver benefits to patients than a pharmacist working in isolation on a medication review (i.e. examining patients' clinical records and making recommendations to the GP at a later date in writing or face-to-face).

The first medication review meetings will take place at the care homes approximately four weeks after allocation to treatment and a second medication review will take place at the 6 month point after allocation to treatment. Each medication review meeting will (on average) consider 15 residents and is expected to last for up to two hours. Thus two face-to-face meetings will be necessary for a care home with around 30 residents and more than two will be required for larger care homes.

The medication review meeting is organised by the pharmacy technician from MMT. The technician will liaise with the care home, GP(s) and the clinical pharmacist (MMT) in order to agree a date and time for the meeting(s). As part of the process of consent, GP practices and care homes have agreed to commit staff time to the medication review meetings. The technician is also responsible for the necessary data extraction from GP practices prior to the medication review meeting. This involves generating patient summary data (see below) - information that allows the clinical pharmacist to complete their preparation for the review meeting.

About one month prior to the intervention (or medication review meeting), a PCT MMT pharmacy technician will visit the care home and obtain a list of residents and their associated GP practices, plus ensure relevant blood tests have been requested with district nurses. Two weeks prior to the planned review meeting the technician will obtain a computer-generated patient summary from the GP practices for each resident to be reviewed. This will include details of current medication, past medical history and recent blood tests. The patient summary data is passed to the clinical pharmacist from MMT. As part of their preparation for the medication review meeting, the clinical pharmacist will prepare a pharmaceutical care plan for each care home resident considering a number of factors, including: the indication, need, evidence base and PCT guidelines for each medication and medical problem. The care plan will also consider any interactions, unmet pharmaceutical needs and clinical appropriateness of each medication.

During the review meeting, each resident's care plan and the associated list of recommended actions prepared by the clinical pharmacist will be discussed by the multi-disciplinary team. As the meeting takes place at the care home, the Medicines Administration Record (MAR) Charts can be consulted during the course of the discussion and any discrepancies with the resident care plan will be identified. A joint decision on action plans will be agreed by the clinical pharmacist, GP, care home staff and, where appropriate, the care home resident. The medication review meeting also provides care home staff with the opportunity to discuss with the clinical pharmacist any general issues relating to medicines management at the care home.

After the review meeting, agreed action plans will be implemented by MMT. The pharmacy technician will update GP records and Read Code the medication review. In addition, the technician will provide written details of all agreed medication changes to the care home and dispensing community pharmacist.

For those MMT members who are not as experienced in care home medication reviews, training on study protocols will be provided by senior members of the MMT. Where a clinical pharmacist undertakes a medication review at a care home for the first time, a senior colleague will evaluate the process and provide feedback. Peer support from more experienced colleagues will be available to members of MMT throughout the study.

The medication review is not designed to focus specifically on issues associated with falls, however many of the interventions which are recommended by pharmacists in medication reviews are likely to impact this outcome, as drug related falls are an important cause of morbidity [30]. Examples include: stopping medication which causes confusion (e.g. psychotropic medication) or adjusting medication which improves mobility (e.g. for Parkinson's disease). It is recognised that the falls prevention context is important and that any fall prevention initiatives that are put in place during the study period have the potential to influence the analysis. While it is envisaged that any falls prevention initiatives would be rolled out consistently across treatment and control, members of the Management Group and Trial Steering Committee (see section entitled Support for the study) will advise the research team of any developments pertinent to the conduct of the study.

### Data collection

Data will be collected from intervention and control homes at three points (baseline, 6 months and 12 months) as follows. All patient demographic, medical history, medication details and falls data (in previous six months) will be obtained from records in the care home (i.e. individual care plans and accident books) and GP practices by a pharmacy technician one month prior to the planned care home visit in intervention homes. For the control homes, data collection at baseline will take place as soon as possible after allocation (within a month of allocation) and will be facilitated by the pharmacy technician. The data collected at this point will include patient demographic and falls data.

Details of all recommendations arising from the medication review, as well as implemented interventions, will be recorded for intervention homes. This process will be repeated after six months. After 12 months follow-up this data will again be extracted from care home records and GP practices (for intervention and control homes, with data collection for the latter encompassing the 6 month and 12 month points) by a pharmacy technician. Anonymised data will be passed to the research team for analysis.

For the economic evaluation, data will be collected on the time taken to develop and provide the intervention and on the resource items likely to change as a result of the intervention. Resource items will include visits to (and from) health care professionals (such as GP, nurse and dietician), as well as hospital and social services.

### Outcome measures

The following primary outcome measures are being utilised:

▪ Number of falls (mean per patient per month)

▪ Potentially inappropriate prescribing (number of drugs which match the STOPP criteria [28] at each data collection point).

Secondary outcome measures include:

▪ Medication costs (mean drug costs per patient - net ingredient costs for 28 days)

▪ Utilisation of primary care, secondary care and personal social services health professional time (GP, nurse and other)

▪ Emergency hospital admissions and Accident and Emergency (A&E) visits (number of admissions in six months per patient)

▪ Mortality.

### Support for the trial

In terms of infrastructure to support the trial and ensure its completion, there are a number of mechanisms in place. In the first instance, the trial is supported by input from a multi-disciplinary research team. In addition to the Principal Investigator (PI), study coordinator and senior pharmacists from the MMT, the team is comprised of specialists in trial design, pharmacy practice, statistics and health economics.

A Management Group will meet on a quarterly basis throughout the duration of the trial. The Management Group consists of the members of the research team, a local GP representative, a community geriatrician, a falls specialist, care home representative and two lay members. The purpose of the Management Group is to set objectives, agree timelines, monitor progress and facilitate study delivery and evaluation.

The study also has a Trial Steering Committee to provide overall supervision of the trial, with an emphasis on trial progress, adherence to protocols, consideration of new information pertinent to the trial, data monitoring, reporting and ethical issues. The Steering Committee is chaired by a statistician and has care home and PCT expert representatives and two lay members. The PI and study coordinator are also members of the Steering Committee.

### Clinical analysis

The null hypothesis is that there will be no difference in primary outcomes between intervention and control patients. All data will be stored on a relational database, which will assist with ongoing management. Data will then be arranged for further analysis in other programs including Microsoft Excel^©^, GenStat and SPSS^©^. The patient population will be characterised using the appropriate descriptive statistics, with medicines categorised according to those described within the British National Formulary (BNF) and medical problems classified according to the World Health Organisation International Classification of Diseases (ICD-10). Descriptive analysis of pharmaceutical care issues and action points in intervention patients will be recorded.

In this paper we are restricting ourselves to a description of our primary analysis, which is a direct comparison of the two groups: intervention and control.

For the following outcome variables - number of falls, emergency hospital admissions and deaths - the total number of events in each care home will be aggregated, along with total resident days 'at risk' in the same period. The events data will be analysed by fitting a hierarchical generalized linear mixed model to them. The basic generalized linear model used will be a log-linear model with Poisson error and log ('at risk' days) as an offset. The care homes themselves will be represented as random effects. Fixed effects in the model will be the intervention itself, the home size (small, medium, large), and resident-type (a two-level factor: residential care vs nursing care). The fact that 'mixed' homes have both types of resident means that the event-counts and 'at risk' days counts will have to be aggregated separately for each type in these homes. The standard error associated with the estimate of the effect of the intervention will be adjusted as necessary to take account of the cluster design. These models will be fitted in the GenStat statistical package using residual maximum likelihood (REML).

It is to be noted that the above analyses are essentially at the level of the care home rather than the patient. There are no identified covariates for the primary analysis. However, exploratory analyses may be conducted at the patient level, should a case emerge for including patient-level covariates in a secondary analysis.

With respect to the study's second primary outcome variable measure (number of drugs which match the STOPP criteria for each patient at each time point), this will also be analysed using a generalized linear mixed model. In this case there is a strong candidate for a patient-level covariate, ie the number of drugs which match the STOPP criteria at baseline. This variable will be analysed at the patient-level, with the baseline covariate (ie the number of drugs which match the STOPP criteria at baseline). The basic generalized linear model here will use a negative binomial distribution for the error. The fixed and random effects will be as previously stated.

### Economic analysis

The economic evaluation will estimate the cost-effectiveness of a MMRS in care homes for older people compared to usual care from a NHS health and personal social services perspective. Established and accepted economic methodologies will be employed throughout [31,32].

An incremental cost analysis will be undertaken. Resource items likely to change as a result of the intervention (as well as the cost of developing and providing the intervention) will be identified, measured and valued. Healthcare resource use data will be collected from care home and GP records. The base case will capture only those costs incurred by the NHS (including, for example, health care professionals' time and travel to medication reviews, patient visits with health care professionals, admissions to secondary care, and medication) and personal social services. Unit costs will be derived from national published data (for instance Curtis 2009 [33]) for the most recent price year available.

This study will undertake a cost-effectiveness analysis to address the technical efficiency question of how best to provide medication review services to older people living in care homes: by multi-professional teams or via GP care (usual care). Effectiveness will be measured in terms of the change in number of falls. If non-dominance occurs (that is if costs are greater and the intervention is more effective or if the intervention is cheaper and less effective) an incremental cost-effectiveness ratio (the ratio of additional cost divided by additional benefit) will be produced in terms of the incremental cost per fall prevented. The confidence region around the incremental cost effectiveness ratio will be estimated using appropriate statistical techniques. Since this economic evaluation is being undertaken alongside a cluster randomised trial the analysis needs to reflect the increased uncertainty of randomising clusters rather than individuals. A number of approaches have recently been proposed for this, with each found to generate similar findings [34]. The stochastic analysis will enable a cost effectiveness acceptability curve to be produced [35] illustrating the decision uncertainty, that is the probability that the MMRS in care homes for older people is cost-effective compared to usual care for a range of willingness to pay per fall prevented values. The timeframe for the economic analysis will be that of the trial period.

### Limitations

The MMRS has the potential to impact on a number of factors that are not being measured in this study, but are likely to be important for medicines management in care homes. For example, the MMRS provides a positive way for pharmacists to work with GPs and care home staff. The review meeting offers an opportunity for the professionals to build relationships and to achieve a greater understanding of their respective roles and responsibilities. The MMRS may also influence positively the medicines culture at the care home, by addressing generic issues in the management and administration of medicines. Further research could explore such factors perhaps utilising action research or other qualitative methodologies. The study is also not measuring the impact of the intervention on the generic health-related quality of life of residents.

## Discussion

In January 2010 the Department of Health issued a CAS Alert [31] calling for a safety review of all aspects of the provision of medication to older people in care homes. Alert (2010) 001 was issued following the publication of the Care Homes Use of Medicines (CHUMS) [32] study that identified a high prevalence of medication errors. The alert calls for an integrated approach to determine how medicines management in care homes might be improved.

This study is seeking to establish the effectiveness of a novel model of clinical medication review in care homes involving a multi-professional approach. It is the first study to undertake an examination of direct patient outcomes together with an economic evaluation. The clinical medication review is innovative in that it involves the pharmacist working in collaboration with GPs and care home staff.

The study also has novel approaches to recruitment and consent. Care homes are being recruited with the support and agreement of their GPs and consent for participation in the study is being sought from GPs and care home managers - and not individual residents. This is because the intervention is provided at the level of the care home (i.e. to all residents) as is standard practice for the MMT team when delivering this service. This approach has the advantage of simplifying the process of securing informed consent. However, it does mean that no additional information is obtained from care home residents and, as indicated above under limitations, the study does not measure the impact of the intervention on, for example, residents' health-related quality of life.

The MMRS is intended to deliver improvements in a number of areas, including the quality of prescribing, increased patient monitoring, better health outcomes, and a potential reduction in the number of medications prescribed. Such a service will contribute to optimising therapy, improving patient safety and minimising problems in the management of medicines at care homes.

## Abbreviations

A&E: (Accident and Emergency); BNF: (British National Formulary); CQC: (Care Quality Commission); GP: (General Practitioner); ICD: (International Classification of Diseases); MAR: (Medicines Administration Record); MMRS: (Multi-professional Medication Review Service); MMT: (Medicines Management Team); NCSC: (National Care Standards Commission); NHS: (National Health Service); PCT: (Primary Care Trust); PI: (Principal Investigator); RCT: (Randomised Controlled Trial); REML: (Residual Maximum Likelihood); STOPP: (Screening Tool of Older Persons Prescriptions);

## Competing interests

The authors declare that they have no competing interests.

## Authors' contributions

JD, SA, RH, TS, VW, JW and DW were responsible for the study design and for securing funding. JD is Principal Investigator. SA and VS will manage study staff, organise training and delivery of the intervention. JH is the study coordinator and will assist in implementing the protocol and ensuring study compliance. JW will conduct and supervise the statistical analysis. TS will conduct the health economics analysis. JD and JH drafted the manuscript. All authors read and approved the final manuscript.
